# Gut Microbial Composition, Oxidative Stress, and Immunity in Metabolic Disease: Toward Personalized Interventions

**DOI:** 10.3390/antiox15020175

**Published:** 2026-01-29

**Authors:** Xuangao Wu, Baide Mu, Guanhao Li, Rui Du, Sunmin Park

**Affiliations:** 1 College of Agriculture, Yanbian University, Yanji 133002, Chinamubaide@ybu.edu.cn (B.M.); durui@ybu.edu.cn (R.D.); 2 Key Innovation Laboratory for Deep and Intensive Processing of Yanbian High-Quality Beef (Co-Construction by Ministry and Province), Ministry of Agriculture and Rural Affairs, Yanji 133002, China; 3 Department of Bioconvergence, Hoseo University, Asan 31499, Republic of Korea; 4 Department of Food and Nutrition, Obesity/Diabetes Research Center, Hoseo University, Asan 31499, Republic of Korea

**Keywords:** microbiota stratification, metabolic disease, oxidative stress, immunity, microbial metabolites, personalized therapeutics

## Abstract

This review examines how distinct gut microbial community configurations—characterized by differential enrichment of *Bacteroides, Prevotella*, *Ruminococcus*, *Bifidobacterium*, and *Lachnospira*—may be associated with variations in host redox homeostasis through microbiota-derived metabolites, including short-chain fatty acids, secondary bile acids, and tryptophan derivatives. These compositional patterns represent reproducible features across populations and correlate with differential disease susceptibility in metabolic disorders. While microbial communities exist along compositional continua rather than discrete clusters, stratification based on dominant patterns offers a pragmatic framework for interpreting large-scale microbiome datasets and guiding precision nutrition interventions. Observational evidence suggests *Bacteroides*-enriched communities may associate with pro-inflammatory signatures, whereas *Prevotella*- *Ruminococcus*, *Proteobacteria*, *Bifidobacterium*, and *Lachnospira*-enriched configurations may exhibit anti-inflammatory or antioxidant characteristics in certain populations. However, inter-population variability and species- and strain-level heterogeneity limit generalization. Condition-dependent effects are exemplified by *Prevotella copri*, which demonstrates pro-inflammatory responses in specific settings despite beneficial profiles in others. When dysbiosis compromises intestinal barrier integrity, microbial translocation may amplify chronic oxidative stress and immune activation. We evaluate therapeutic potential of beneficial genera including *Lactobacillus* and *Bifidobacterium* while examining the dose-dependent, context-specific, and sometimes paradoxical effects of key metabolites. Microbiota-stratified therapeutic strategies—personalizing dietary, probiotic, or prebiotic interventions to baseline community composition—show promise but remain at proof-of-concept stage. Current evidence derives predominantly from cross-sectional and preclinical studies; prospective interventional trials linking community stratification with oxidative stress biomarkers remain scarce. The community–redox relationships presented constitute a hypothesis-generating framework supported by mechanistic plausibility and observational associations, rather than established causal pathways. Future research should prioritize intervention studies assessing whether aligning therapeutic approaches with baseline microbial configurations improves outcomes in oxidative stress-related metabolic disorders.

## 1. Introduction

Oxidative stress—an imbalance between reactive oxygen species (ROS) generation and endogenous antioxidant defenses—is a central mechanism linking metabolic syndrome, metabolic dysfunction-associated steatotic liver disease (MASLD), cardiovascular disease (CVD), and obesity [1,2,3,4]. This review focuses on oxidative stress within the intestinal environment and along the gut–liver and systemic axes, where microbial metabolites, epithelial barrier function, and immune responses intersect.

The gut microbiota is a key modulator of host redox homeostasis and immune programming. Although endogenous antioxidant systems such as superoxide dismutase, catalase, and glutathione peroxidase are robust, they may become insufficient under chronic metabolic stress, inflammation, or dysbiosis [5]. Human gut microbial communities exhibit reproducible compositional patterns that can be stratified based on dominant taxa—including communities enriched in *Bacteroides*, *Prevotella*, *Ruminococcus*, *Bifidobacterium*, *Proteobacteria*, or *Lachnospira*—which differ in their metabolic functions and redox-regulatory capacities [6,7]. These microbial community configurations exist along compositional continua rather than discrete clusters, yet stratification based on dominant patterns provides a pragmatic framework for organizing and analyzing large-scale microbiome datasets, particularly in precision nutrition research. Specific microbiota compositions have been linked to the prevalence of metabolic diseases across populations. *Proteobacteria*-dominant communities, such as those high in Escherichia, are more strongly associated with disease but are not included in this review due to their low prevalence and limited population-level data.

In *Bacteroides*-enriched communities, elevated levels of lipopolysaccharide (LPS)-containing Gram-negative bacteria may engage Toll-like receptor 4 (TLR4) signaling and promote pro-inflammatory immune activation, although importantly, *Bacteroides*-derived LPS is less immunogenic than that of *Escherichia coli* [8]. These communities also generate acetate and propionate via carbohydrate fermentation, highlighting context-dependent metabolic effects. *Prevotella*-enriched configurations typically align with fiber-rich diets and higher fluxes of short-chain fatty acids (SCFA), which can activate anti-inflammatory pathways and mitigate oxidative stress. However, certain *Prevotella* species, notably *Prevotella copri (P. copri*), can be pro-inflammatory depending on diet, host genetics, and inflammatory conditions [9].

*Ruminococcus*- and *Lachnospira*-enriched profiles are characterized by butyrate-producing taxa, which confer antioxidant and anti-inflammatory effects partly through sirtuin activation pathways [10]. Other beneficial microbes, including *Lactobacillus* and *Bifidobacterium*, possess intrinsic antioxidant mechanisms—superoxide dismutase, catalase, and exopolysaccharide production—that neutralize ROS and promote anti-inflammatory immune responses [11,12]. Community configuration-associated differences in bioactive metabolites—including SCFAs, secondary bile acids, tryptophan derivatives, and gasotransmitters such as hydrogen sulfide—create distinct biochemical environments that modulate oxidative stress, immune activation, and metabolic signaling [13,14,15].

However, it is important to emphasize that direct causal links between microbial community classifications and oxidative stress outcomes remain largely inferred rather than definitively established. Most available evidence derives from cross-sectional associations, short-term interventional studies, or preclinical models, with limited longitudinal or mechanistic data directly connecting community composition states to changes in oxidative stress biomarkers or clinical endpoints in humans. Given that microbiota-targeted interventions often yield inconsistent outcomes due to inter-individual variation in host genetics, diet, medication use, and baseline microbiota composition, there is growing interest in more stratified, microbiota-informed therapeutic approaches. This review synthesizes current evidence on how distinct microbial community configurations and their metabolites may influence the redox–immune axis in metabolic diseases, examines the therapeutic potential of microbiota modulation strategies, and proposes a conceptual framework to guide hypothesis-driven research and the development of precision microbiome-based interventions for the prevention and treatment of metabolic diseases.

## 2. Interplay Between Oxidative Stress, Redox Homeostasis, and the Immune System

### 2.1. Molecular Basis of Redox Homeostasis and Endogenous Antioxidant Systems

Redox reactions promote fundamental cellular processes and are dynamically controlled to maintain homeostasis [16]. Environmental and microbial cues generate ROS and reactive nitrogen species (RNS) that reversibly modify protein cysteines and reshape redox signaling networks [17]. A central sensor is the Keap1–nuclear factor erythroid 2-related factor 2 (Nrf2) axis: Keap1-mediated degradation keeps Nrf2 low at baseline, whereas ROS-induced Keap1 oxidation releases Nrf2 to drive an antioxidant transcriptional program, including NADPH-dependent glutathione- and thioredoxin-based selenoenzymes [18]. Redox modification of metabolic enzymes such as glyceraldehyde 3-phosphate dehydrogenase (GAPDH) further links this pathway to metabolic flux reprogramming and cellular stress tolerance [19].

Commensal communities ferment dietary fibers into short-chain fatty acids, particularly butyrate, which not only fuels epithelial mitochondrial metabolism but also can enhance antioxidant defenses, such as through activation of Nrf2-related pathways, and help maintain barrier integrity, thereby limiting mucosal ROS accumulation and oxidative damage [20,21].

### 2.2. The Oxidative-Stress–Immunity Axis: NF-κB and NADPH Oxidase Pathways

Oxidative stress functions as a central immune signal that modulates inflammation, cell fate decisions, and immune homeostasis [22]. Recognition of bacterial LPS by TLR4 activates myeloid differentiation primary response 88 (MyD88)- and TIR-domain-containing adapter-inducing interferon-β (TRIF)-dependent cascades, leading to inhibitor of κB (IκB) degradation, nuclear factor κB (NF-κB) nuclear translocation, and the induction of cytokines such as tumor necrosis factor (TNF)-α, interleukin (IL)-1β, and IL-6, as well as NADPH oxidase (NOX) family members [23]. NOX-derived superoxide (O_2_•^−^) and hydrogen peroxide (H_2_O_2_) amplify these pathways as second messengers but, when produced chronically, cause lipid peroxidation, protein and DNA damage, ultimately impairing immune cell function [19,22]. NF-κB-driven inflammation and the Keap1–Nrf2 antioxidant system therefore form an antagonistic pair: NF-κB promotes iNOS/NOX activity and suppresses Nrf2-dependent cytoprotective programs, whereas Nrf2 activation limits ROS accumulation and dampens NF-κB signaling. The dynamic balance between the TLR4–NF-κB–NOX inflammatory axis and the Keap1–Nrf2 pathway is thus critical for appropriate immune activation.

In the intestinal mucosa, this balance is strongly influenced by the gut microbiota. Dysbiosis increases exposure to LPS and other microbial ligands that activate TLR4–NF-κB–NOX signaling and drive oxidative stress. Commensal-derived metabolites such as butyrate can activate Nrf2, strengthen the epithelial barrier, and attenuate ROS-driven inflammation [24,25].

### 2.3. Oxidative Stress as a Driver of Chronic Inflammation and Metabolic Disease

The reciprocal relationship between oxidative stress and chronic inflammation creates self-perpetuating cycles that drive the pathogenesis of metabolic disease. ROS activate NF-κB and other redox-sensitive pathways, inducing pro-inflammatory cytokines such as IL-1β, IL-6, and TNF-α. Neutrophils and macrophages at inflamed sites further aggravate oxidative stress by generating ROS via NOX enzymes [26,27,28]. This amplification loop is evident not only in autoimmune conditions such as rheumatoid arthritis (RA) but also in metabolic disorders, where hyperglycemia increases mitochondrial ROS through protein kinase C activation, hexosamine pathway flux, and advanced glycation end product (AGE) formation [29,30]. ROS directly impair pancreatic β-cell function and reduce insulin secretion. In adipose tissue, the combination of oxidative stress and chronic low-grade inflammation promotes insulin resistance via TNF-α/IL-6–driven serine/threonine phosphorylation of insulin receptor substrate (IRS)-1, thereby disrupting insulin signaling [28].

Mitochondrial dysfunction represents a critical nexus linking oxidative stress, chronic inflammation, and metabolic disease. As major intracellular sources of ROS, damaged mitochondria intensify oxidative stress and release damage-associated molecular patterns (DAMPs), such as mitochondrial DNA (mtDNA), that engage innate immune sensors including cyclic GMP–AMP synthase–stimulator of interferon genes (cGAS–STING) and TLR9 to trigger inflammatory responses [31,32,33]. Preserving mitochondrial homeostasis is therefore crucial for preventing and controlling metabolic diseases.

In the intestine, similar oxidative–inflammatory cycles develop at the mucosal surface, where dysbiosis and barrier disruption increase exposure to microbial products that activate TLRs and inflammasomes, driving mitochondrial ROS production and epithelial damage [25]. Conversely, several commensal-derived metabolites, including butyrate and indole derivatives, have been shown to enhance mitochondrial function, upregulate antioxidant pathways, and strengthen tight junctions, thereby attenuating gut inflammation and systemic metabolic disturbances [20].

## 3. Microbial Community Stratification and the Gut Microbiota–Redox–Immune Axis

**Figure 1:**
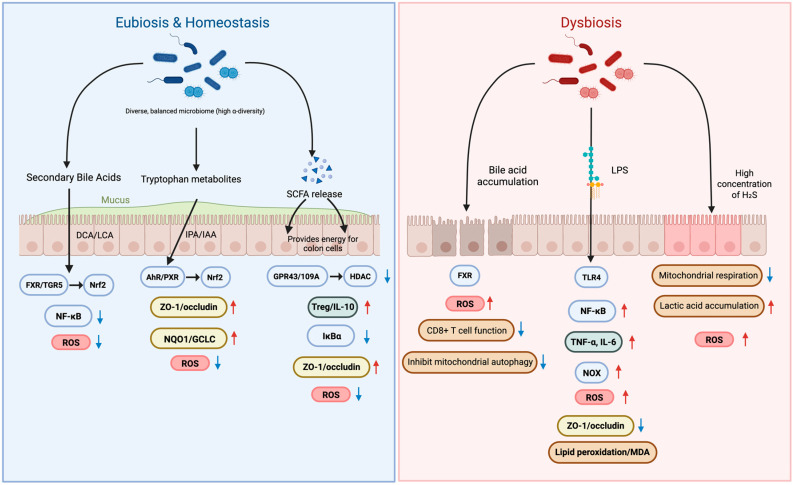
Microbial Metabolites in Eubiosis vs. Dysbiosis: Impact on Oxidative Stress and Barrier Integrity. Red upward arrows denote increased levels, whereas blue downward arrows denote decreased levels.

The gut microbiota influences host oxidative stress and inflammation, but these effects vary across individuals and depend strongly on overall community structure. In states of eubiosis, a functionally diverse microbiota generates metabolites, including SCFAs, secondary bile acids, and indole derivatives, that maintain epithelial barrier integrity and modulate immune thresholds. Conversely, dysbiosis leads to the accumulation of pro-oxidant and pro-inflammatory molecules such as LPS and excess H_2_S, amplifying oxidative stress and inflammatory cascades (Figure 1).

Gut microbial communities exhibit reproducible compositional patterns characterized by differential enrichment of key taxa, including *Bacteroides*, *Prevotella*, *Ruminococcus*, *Bifidobacterium*, and *Lachnospira* [34]. However, these communities exist along compositional gradients rather than as discrete categories. Individuals often fall along continua defined by carbohydrate-fermenting versus protein-fermenting taxa, and many populations show mixed or intermediate configurations, with substantial intra-individual variation over time and across populations. Long-term dietary patterns correlate strongly with these community states [35], and short-term dietary interventions can rapidly shift microbial composition: animal-based diets increase bile-tolerant taxa (*Alistipes*, *Bilophila*, *Bacteroides*), whereas plant-based diets favor Firmicutes that metabolize polysaccharides (*Roseburia*, *Eubacterium rectale*, *Ruminococcus bromii*) [36]. While community boundaries remain indistinct and classification methods continue to be refined, stratification frameworks based on dominant compositional patterns remain pragmatic tools for organizing large-scale microbiome data and generating hypotheses about metabolic capacities linked to specific community configurations [35,36]. Community-type designations capture meaningful differences in metabolic capacity—particularly regarding carbohydrate fermentation, SCFA production profiles, and bile acid metabolism—that correlate with dietary patterns and metabolic phenotypes across diverse cohorts. In this review, microbial community configurations are used as analytical archetypes that enrich for specific metabolic and immunomodulatory capacities rather than as rigid taxonomic boundaries. This approach aligns with emerging precision nutrition strategies, where baseline microbial community structure informs personalized dietary and therapeutic interventions [37]. Below, key taxa and metabolites enriched across different community configurations are summarized, and evidence linking them to redox and immune processes is critically evaluated. These patterns represent tendency clusters along a compositional continuum, and individual-level effects depend heavily on species- and strain-level composition, diet, host genetics, and metabolic context. Importantly, direct causal evidence linking community configuration states to oxidative stress or clinical outcomes remains limited, heterogeneous across cohorts, and often confounded by nutrition, ethnicity, or medication exposure.

This figure illustrates how gut microbial metabolites regulate oxidative stress and intestinal barrier integrity by contrasting eubiosis and dysbiosis. In eubiosis, a diverse microbiome produces beneficial metabolites like secondary bile acids, tryptophan metabolites, and SCFAs. These compounds activate signaling pathways such as FXR and AhR that enhance tight junctions (ZO-1/occludin) and reduce oxidative stress by downregulating ROS. This maintains a strong, healthy intestinal barrier. In contrast, dysbiosis—an imbalanced microbiome—is characterized by the accumulation of harmful substances like LPS, which activate inflammatory pathways such as TLR4 and NF-κB. This leads to increased oxidative stress, breakdown of the intestinal barrier, and heightened inflammation (TNF-α, IL-6), ultimately contributing to conditions like “leaky gut” and cellular damage. SCFAs, short-chain fatty acids; DCA, deoxycholic acid; LCA, lithocholic acid; IPA, indole-3-propionic acid; IAA, indole-3-acetic acid; ROS, reactive oxygen species; FXR, farnesoid X receptor; TGR5 (GPBAR1), G-protein-coupled bile acid receptor 1; AhR, aryl hydrocarbon receptor; PXR, pregnane X receptor; HDAC, histone deacetylase; SIRT1, sirtuin 1; IκBα, inhibitor of NF-κB; TLR4, toll-like receptor 4; NOX, NADPH oxidase; MDA, malondialdehyde; ZO-1, tight junction protein-1, TNF-α, tumor necrosis factor alpha; IL-6, interleukin-6.

### 3.1. Microbial Composition and Metabolic Modulation

Different gut microbial community configurations exhibit distinct metabolic capacities that influence host redox and immune homeostasis. Below we summarize key compositional patterns and their associated metabolic characteristics, recognizing these represent tendencies along compositional continua rather than discrete categories.

#### 3.1.1. *Bacteroides*-Enriched Communities: LPS and Acetate/Propionate Production

*Bacteroides*-dominant communities generate acetate and propionate from complex carbohydrates and contribute substantially to total gut LPS mass [38]. However, *Bacteroides* LPS is structurally less endotoxic than Enterobacteriaceae LPS, which is a strong TLR4 agonist that drives NF-κB activation, pro-inflammatory cytokine release (TNF-α, IL-1β, IL-6), and NOX-dependent ROS production [39,40,41,42,43]. The inflammatory or oxidative potential of *Bacteroides*-enriched configurations depends less on *Bacteroides* itself and more on the accompanying microbial context, particularly the abundance of high-potency LPS-producing pathobionts. Although several cohort studies have reported associations between *Bacteroides*-dominant profiles and metabolic dysfunction, findings remain inconsistent [10,44] and often lose significance after adjustment for dietary fat intake, antibiotic use, or host metabolic factors.

#### 3.1.2. *Prevotella*-Enriched Communities: Condition-Dependent SCFA Production

*Prevotella*-enriched communities are strongly associated with fiber-rich, carbohydrate-dominant dietary patterns, reflecting advanced capacity to utilize complex polysaccharides [45,46,47]. These microbes efficiently ferment dietary fibers to produce SCFAs—primarily acetate and propionate, with modest butyrate levels. These communities tend to exhibit anti-inflammatory or antioxidant features through SCFA-mediated signaling via GPR43 and GPR109A, which restrains NLRP3 inflammasome activation and improves barrier function [48,49]. Additionally, butyrate and propionate inhibit histone deacetylases (HDACs), promoting Foxp3+ regulatory T cell differentiation and IL-10 pathway activation.

However, effects are highly context-sensitive. Under concurrent TLR stimulation, HDAC inhibition may paradoxically facilitate rather than suppress NLRP3 inflammasome activation [50]. Moreover, specific species can reverse typical benefits: *P. copri* expansion can synergize with high-fiber intake to exacerbate inflammation in genetically predisposed individuals through over-fermentation to succinate and fumarate—metabolites that activate pro-inflammatory Th17 pathways [51,52]. These dual characteristics underscore that *Prevotella*-enriched communities are not intrinsically protective or harmful, but rather condition-dependent configurations whose net impact depends on species-level composition, host immunogenetics, and diet.

#### 3.1.3. Ruminococcaceae/Lachnospiraceae-Enriched Communities: Butyrate-Sirtuin Axis

Communities enriched in Ruminococcaceae and Lachnospiraceae species, particularly those with high abundance of *Faecalibacterium prausnitzii*, are characterized by elevated butyrate production [53]. These taxa frequently engage in cross-feeding interactions, converting acetate (largely supplied by Bifidobacterium) into butyrate, resulting in high colonic butyrate availability [54]. This metabolic specialization is thought to support epithelial health and more favorable systemic metabolic profiles. Butyrate serves as the primary energy source for colonocytes, enhancing mitochondrial oxidative metabolism and oxygen consumption [55]. Butyrate serves as the primary energy source for colonocytes, and its oxidation shifts cellular NAD+/NADH ratios, activating SIRT1 [56,57]. Active SIRT1 deacetylates NF-κB p65 to suppress inflammatory signaling while upregulating antioxidant enzymes (MnSOD, catalase) through PGC-1α and FOXO transcription factors.

Multi-cohort studies report associations between these community patterns and reduced metabolic syndrome risk [37]. However, these configurations remain less extensively characterized than *Bacteroides*- or *Prevotella*-enriched communities, and their hallmark butyrate producers are widely distributed across different community types, complicating attribution of effects to community-level versus species-level features.

### 3.2. Microbial Metabolites and Oxidative Stress

The key microbial metabolites that may mediate the gut microbiota–redox–immune axis in metabolic diseases are summarized in Table 1.

#### 3.2.1. SCFAs and Immune Modulation

SCFAs, particularly butyrate, serve dual roles as both essential metabolic substrates and potent signaling molecules in the gut microbiota–redox–immunity axis [9,74]. Their metabolic functions enhance cellular bioenergetics by elevating oxidative phosphorylation capacity and sparing respiratory reserve, thereby strengthening energy and redox buffering capabilities under stress conditions and reducing ROS-related cellular injury [75,76]. As signaling molecules, SCFAs function as endogenous ligands for G-protein-coupled receptors GPR43 and GPR109A, initiating cascades that stabilize IκBα, limit NF-κB (p65) nuclear translocation, and repress the production of inflammatory cytokines [77,78]. Simultaneously, SCFAs activate AMP-activated protein kinase (AMPK)–Nrf2 signaling pathways, promoting Nrf2 nuclear translocation and target gene expression, thereby coupling anti-inflammatory pathways with enhanced antioxidant responses. This dual signaling mechanism creates a functional hub that links energy metabolism with mucosal barrier integrity and immune homeostasis.

The protective effects extend beyond immediate signaling to include support for epithelial cell fueling and mucus synthesis. By tuning the NF-κB/Nrf2 balance, SCFAs effectively set innate immune response thresholds to both internal and external oxidative challenges [79,80]. This coordinated regulation of metabolic fueling plus transcriptional reprogramming reduces system-level transitions from inflammatory activation to oxidative tissue damage.

#### 3.2.2. Secondary Bile Acids and Oxidative Stress

The bile acid system represents a sophisticated liver-gut-microbe relay that generates highly active signaling pools through sequential transformations [81,82,83,84]. Primary bile acids synthesized in the liver undergo conjugation before entering the gut, where microbial bile salt hydrolase (BSH) enzymes deconjugate them. Subsequently, gut bacteria progress further transformations, including dehydrogenation, dehydroxylation, oxidation, and epimerization, creating diverse secondary bile acid species with distinct biological activities. Within physiological concentration ranges, secondary bile acids exert protective effects by signaling through the Farnesoid X Receptor (FXR) and Takeda G protein-coupled receptor 5 (TGR5) pathways [85,86,87]. This signaling suppresses NF-κB activation, dampens lipid peroxidation and ROS production, and ameliorates diet-induced metabolic derangements. However, the bile acid-redox axis is highly context-dependent in its effects. Dysregulation of this system can have adverse consequences: disruption of the FXR pathway or pathological accumulation of specific bile acid species can trigger endoplasmic reticulum stress and ROS bursts, suppress CD8+ T cell function, and inhibit mitophagy processes [88,89,90]. When mitophagy is impaired, damaged mitochondria accumulate, leading to increased ROS production and cellular dysfunction.

#### 3.2.3. Tryptophan-Derived Metabolites and Redox Signaling

Dietary tryptophan metabolism branches into several interconnected pathways with major implications for redox homeostasis, epithelial integrity, and immune regulation. These include: (1) the microbial indole pathway; (2) the host kynurenine pathway; and (3) the serotonin (5-HT)/melatonin axis [91,92]. Microbial indole derivatives—including indole-3-propionate (IPA), indole-3-acetate (IA), and indole-3-carboxaldehyde (I3C)—serve as endogenous ligands for the aryl hydrocarbon receptor (AhR) and pregnane X receptor (PXR). Activation of these receptors induces detoxification and antioxidant response programs, including Nrf2-related gene networks, enhances epithelial barrier function, and dampens inflammatory and oxidative signaling [93,94,95]. IPA, a particularly potent PXR ligand, provides a direct microbe-to-host signaling pathway that links microbial metabolism to host redox regulation. Beyond receptor signaling, IPA also exhibits direct radical-scavenging effects and mitochondrial protection in neuronal and cardiomyocyte models. However, both AhR activation and indole activity are dose- and context-dependent; excessive or dysregulated ligand exposure may shift responses toward pro-oxidant or pro-inflammatory states [95].

The kynurenine pathway is the only de novo source of NAD^+^ from dietary tryptophan in mammals, thus directly linking amino acid metabolism to cellular energy and redox status [96]. Metabolites within this pathway exert divergent effects: 3-hydroxykynurenine (3-HK) and quinolinic acid (QUIN) are generally pro-oxidant and excitotoxic, whereas kynurenic acid (KYNA) has radical-scavenging and neuroprotective actions. The balance of these metabolites—shaped by enzyme activities such as kynurenine 3-monooxygenase (KMO) and kynurenine aminotransferases (KATs)—helps define tissue-specific oxidative environments [97]. Potential therapeutic approaches include enhancing beneficial indoles (particularly IPA), inhibiting KMO, and augmenting NAD^+^ synthesis, though clinical translation will require careful dose titration and consideration of inter-individual variation in receptor sensitivity, microbial composition, and baseline oxidative states [98,99,100,101,102].

Notably, SCFAs also interact with the mucosal serotonergic system, adding the layer of metabolic–immune–redox integration. Butyrate and related SCFAs upregulate tryptophan hydroxylase-1 (TPH1) in enterochromaffin cells and increase colonic 5-HT synthesis and release, thereby linking microbial fermentation of dietary fiber to local serotonin availability. Through this SCFA–5-HT axis, microbial metabolites and host-derived 5-HT jointly calibrate mucosal immune responses, epithelial barrier function, and local oxidative stress regulation [79].

#### 3.2.4. Gaseous Transmitter (H_2_S) and Antioxidant Actions

Sulfur metabolism occupies a pivotal position in the microbiota-host redox network, with cysteine serving as both the rate-limiting substrate for glutathione (GSH) synthesis and a key determinant of thiol redox modification dynamics [103,104]. Dysbiotic conditions can significantly reduce plasma glutathione peroxidase activity and GSH levels, thereby amplifying systemic susceptibility to oxidative stress. Some gut microbial species produce H_2_S through trans-sulfuration pathways, establishing this gaseous molecule as an important microbiota-derived signaling mediator [105]. The biological effects of microbial H_2_S demonstrate remarkable dose- and situation-dependency. At physiological concentrations, H_2_S maintains beneficial protein S-sulfhydration modifications, supports iron-sulfur cluster biogenesis essential for numerous enzymes, enhances mitochondrial energetics, and upregulates glutathione synthesis pathways—combining antioxidant effects and cellular repair [104,106].

However, excessive H_2_S production or impaired clearance mechanisms can trigger severe pathophysiological consequences. High H_2_S concentrations inhibit cytochrome c oxidase (complex IV), causing lactate accumulation and adenosine triphosphate (ATP) depletion [107]. When sulfide oxidation pathways such as sulfide quinone oxidoreductase become deficient, H_2_S accumulation can lead to severe neuropathology. Additionally, excess H_2_S disrupts normal networks of cysteine and glutathione redox modification, including the glutathionylation and deglutathionylation cycles, triggering protein misfolding and ferroptotic cell death pathways. Protective effects emerge from fine-tuned thiol signaling and mitochondrial respiratory support. Toxicity occurs due to inhibiting the respiratory chain and disrupting the redox signaling network. Understanding this balance proves essential for therapeutic approaches targeting sulfur metabolism in the gut microbiome-host interface [108,109,110,111,112].

## 4. The Microbiota–Redox–Immune Axis in the Pathophysiology of Metabolic Diseases

The gut microbiota–redox–immune axis represents a core mechanistic pathway implicated in the development and progression of metabolic diseases. Disturbances in this axis arise from mutually reinforcing processes involving epithelial barrier dysfunction, oxidative stress amplification, and chronic immune activation. These maladaptive cycles contribute to the pathophysiology of conditions such as metabolic syndrome, obesity, MASLD, and type 2 diabetes (T2DM) [10,113].

Across multiple metabolic and inflammatory disease cohorts, *Bacteroides*-dominant communities—particularly low-diversity *Bacteroides*-enriched configurations (Bact2 subtype)—are disproportionately represented and consistently correlate with heightened inflammatory load, impaired barrier integrity, and adverse metabolic profiles [114]. This enrichment of *Bacteroides*-dominant community structures is associated with a shift toward pro-inflammatory metabolites, increased luminal LPS activity, and reduced production of protective microbial metabolites such as butyrate. In contrast, *Prevotella*-enriched profiles tend to be proportionally reduced in most metabolic disease cohorts, although this pattern is not universal. Notably, *Prevotella*-enriched communities appear more frequently in colorectal neoplasia, where specific *Prevotella*-rich configurations have been associated with colorectal adenoma and early tumorigenesis [115,116,117]. These findings highlight that the pathogenic significance of a community configuration is condition-dependent and shaped by species-level composition, disease environment, and host factors. This section examines how dysbiotic microbial communities—through altered metabolite profiles, redox imbalance, and immune dysregulation—may contribute to the initiation and perpetuation of metabolic disease.

### 4.1. T2DM and Insulin Resistance

The progression from metabolic health to T2DM involves systematic perturbations of the microbiota–redox–immune axis to create self-reinforcing pathological cycles. Across the spectrum from prediabetes to established diabetes, characteristic microbiome alterations include reduced microbial diversity, depletion of beneficial butyrate-producing bacteria, particularly *F. prausnitzii* and *Akkermansia muciniphila* (*A. muciniphila*), and expansion of conditional pathobionts such as Enterobacteriaceae and *Desulfovibrio* species [118].

#### 4.1.1. Mechanistic Pathways Linking Dysbiosis to Insulin Resistance

The pathophysiological cascade begins with diet-induced dysbiosis, particularly under conditions of high-fat and high-sugar diets. Dysbiotic communities increase intestinal permeability through the degradation of tight junction proteins and thinning of the mucus layer, facilitating the translocation of LPS and other microbial antigens into the systemic circulation—a condition termed metabolic endotoxemia [119]. Circulating LPS activates TLR4 on immune cells and metabolic tissues, triggering NF-κB-mediated inflammatory cascades that produce pro-inflammatory cytokines, including TNF-α, IL-1β, and IL-6. This inflammatory activation directly impairs insulin signaling through multiple mechanisms. Excess ROS generated by NADPH oxidase and mitochondrial dysfunction inhibit insulin receptor substrate-1 (IRS-1) and phosphoinositide 3-kinase (PI3K)/protein kinase B (Akt) signaling pathways, while inflammatory cytokines induce serine phosphorylation of IRS-1, further blocking insulin sensitivity in muscle and liver tissues [120]. The importance of this pathway is demonstrated by studies showing that TLR4-deficient mouse models resist diet-induced obesity and insulin resistance, thereby supporting a causal relationship between microbial sensing and metabolic dysfunction in experimental models [119].

#### 4.1.2. Pancreatic β-Cell Dysfunction and Oxidative Stress

Beyond peripheral insulin resistance, the microbiota–redox–immune axis plays a critical role in influencing pancreatic β-cell function. Chronic oxidative stress damages β-cell mitochondria, impairing glucose-stimulated insulin secretion and promoting β-cell apoptosis [120]. The NLRP3 inflammasome, activated by obesity-associated dysbiosis, couples IL-1β-mediated β-cell suppression with peripheral insulin resistance, creating a bidirectional pathological relationship between pancreatic dysfunction and systemic metabolic impairment [121].

#### 4.1.3. Protective Mechanisms and Therapeutic Targets

SCFAs play a significant role in arresting the progression of diabetes. Under hyperglycemic and inflammatory conditions, SCFAs act through GPR43 and β-arrestin-2 signaling to suppress NF-κB activation, enhance the gene expression of antioxidant enzymes, and amplify Nrf2-mediated protective responses [77]. This alleviates oxidative stress in renal and pancreatic tissues while improving insulin signaling in preclinical models. Additionally, SCFAs promote incretin secretion, enhance β-cell function, and improve glucose homeostasis through activating enteroendocrine cells. The therapeutic implications are supported by interventional studies showing that microbiota-targeted approaches, including dietary fiber supplementation, probiotic administration, and fecal microbiota transplantation (FMT), can improve glycemic control and reduce inflammatory markers in patients with diabetes. These findings support the microbiota–redox–immune axis as both a potential pathogenic driver and therapeutic target in T2DM management.

### 4.2. MASLD

MASLD represents a paradigmatic example of gut–liver axis dysfunction, in which intestinal dysbiosis is positively associated with hepatic injury through inflammatory and oxidative mechanisms [122]. Dysbiosis impairs the intestinal barrier and increases portal influx of LPS, secondary bile acids, and other microbial products, activating hepatic Kupffer cells via TLR4, upregulating TNF-α and IL-6, and driving sustained ROS generation and lipid peroxidation [123]. Oxidized lipid products such as malondialdehyde and 4-hydroxynonenal act as DAMPs, further amplifying NF-κB signaling and chronic inflammation. In parallel, dysregulated bile acid metabolism—reduced BSH activity, accumulation of hydrophobic secondary bile acids, and impaired FXR signaling—contributes to stellate cell activation, fibrosis, and disturbances in lipid and glucose metabolism [124]. Hepatic ROS excess disrupts IRS–PI3K signaling and directly damages pancreatic β-cells, promoting systemic insulin resistance and reduced insulin secretion, thereby reinforcing the bidirectional relationship between MASLD and T2DM [125,126].

Microbial metabolites act as double-edged modulators of MASLD progression. Under physiological conditions, SCFAs, particularly butyrate, can attenuate hepatic oxidative stress, enhance antioxidant enzyme activity, and promote fatty acid oxidation while limiting de novo lipogenesis through AMPK- and mitochondria-dependent mechanisms [127]. By contrast, excessive production of potentially harmful metabolites such as H_2_S and trimethylamine N-oxide (TMAO) may worsen hepatic dysfunction: high H_2_S levels can suppress glucagon-like peptide-1 (GLP-1) secretion, impair insulin sensitivity, and increase mitochondrial ROS, whereas elevated TMAO correlates with MASLD severity and promotes macrophage-driven hepatic inflammation [128]. These insights have inspired microbiota-targeted strategies, including prebiotic fiber aimed at stimulating SCFA production, probiotic supplementation to restore beneficial taxa, and bile acid receptor modulators, to support microbiota homeostasis, hepatic redox balance, and metabolic health.

### 4.3. Cardiovascular Disease (CVD) and Obesity

CVD and obesity share core mechanisms within the gut microbiota–redox–immune axis, whereby dysbiosis increases intestinal permeability and metabolic endotoxemia, driving endothelial dysfunction and atherosclerosis through LPS–TLR4 activation, vascular NOX-derived ROS, and mitochondrial oxidative stress [129]. Experimental and clinical data indicate that inhibiting NOX activity preserves nitric oxide (NO) bioavailability, limits vascular remodeling, and confers cardiovascular protection [130].

Community composition patterns appear relevant: the dysbiotic *Bacteroides*-dominant Bact2 subtype is enriched in individuals with elevated inflammatory markers and higher cardiovascular risk, and its prevalence decreases with statin use, suggesting interplay between pharmacological treatments and microbiome configurations [131]. Microbial metabolites further modulate risk in opposing directions: TMAO promotes vascular inflammation, platelet activation, and adverse cardiovascular events [132,133], whereas SCFAs, particularly butyrate and propionate, lower blood pressure, improve endothelial function, and attenuate vascular oxidative stress via GPR41/GPR43 and Nrf2 signaling; prebiotic strategies that enhance SCFA production have shown corresponding improvements in hemodynamic and vascular parameters [9,47,134,135,136,137,138,139,140].

In obesity, dysbiotic communities promote infiltration of adipose tissue macrophages, local oxidative stress, and systemic insulin resistance, thereby amplifying cardiometabolic risk. These insights collectively support microbiota-modulating, antioxidant, and anti-inflammatory interventions as complementary strategies for cardiovascular prevention and treatment.

## 5. Therapeutic Intervention Strategies

The growing understanding of how gut microbiota and microbial stratification-related patterns modulate oxidative stress and immunity, based largely on preclinical studies and a limited number of early clinical trials, has opened the possibility of precision-based therapeutic strategies that modulate gut microbiota composition and function according to individual microbiome profiles and metabolic capacity.

### 5.1. Probiotics and Redox Regulation

Probiotic interventions represent a direct approach to modulating gut microbial balance and host redox homeostasis through metabolite production, activation of host pathways, and enhancement of enzymatic antioxidants. Most mechanistic insights derive from cell and animal studies, whereas early clinical trials in metabolic and inflammatory diseases report modest improvements in oxidative stress markers.

#### 5.1.1. Metabolite-Mediated Redox Effects

Several probiotic strains exert their beneficial effects through the production of bioactive metabolites that directly modulate host redox status. *Limosilactobacillus reuteri (L. reuteri)* produces reuterin (3-hydroxypropionaldehyde), a unique antimicrobial compound that induces controlled oxidative stress specifically targeting pathogenic microorganisms and cancer cells [64]. Reuterin suppresses ribosome biogenesis and protein translation in colorectal cancer cells through depleting glutathione and repressing cysteine modifications. It effectively reprograms cellular redox balance to inhibit tumor growth and extend survival in preclinical models. *F. prausnitzii*, a key butyrate producer frequently depleted in metabolic diseases, exerts protective effects through multiple mechanisms. *F. prausnitzii*-derived extracellular vesicles activate the Nrf2/heme oxygenase-1 (HO-1) pathway while delivering butyrate directly to colonocytes, maintaining intestinal barrier homeostasis and alleviating inflammatory responses [72]. This dual mechanism of antioxidant pathway activation, combined with the delivery of anti-inflammatory metabolites, exemplifies the sophisticated ways by which beneficial bacteria support host health.

#### 5.1.2. Host Pathway Activation

Beyond metabolite production, specific probiotic strains directly activate host antioxidant defense pathways. *Lacticaseibacillus rhamnosus* (*L. rhamnosus*) GG demonstrates remarkable hepatoprotective effects by activating hepatic Nrf2 signaling and upregulating downstream antioxidant genes, including glutathione S-transferases (GSTs), NAD(P)H quinone oxidoreductase 1 (NQO1), and heme oxygenase-1 (HO-1) [62]. This activation protects against drug-induced and alcohol-induced oxidative liver injury, highlighting the therapeutic potential of targeted probiotic interventions in hepatic diseases. Similarly, *Bifidobacterium bifidum* (*B. bifidum*) enhances total antioxidant capacity by activating the Keap1–Nrf2 pathway, with clinical evidence demonstrating improved oxidative stress markers in diabetes patients on hemodialysis who have particularly high oxidative [67]. These findings suggest that specific probiotic strains can serve as biological modulators of host antioxidant capacity.

#### 5.1.3. Enzymatic Detoxification

Many probiotic strains possess intrinsic antioxidant enzymatic systems that directly contribute to ROS detoxification. Multiple *Lactobacillus* and *Bifidobacterium* species, *including L. johnsonii*, *Levilactobacillus brevis MG000874* (*L. brevis*), *Lactiplantibacillus plantarum* (*L. plantarum*), *B. longum*, and *B. breve*, demonstrate enhanced superoxide dismutase (SOD), catalase (CAT), and glutathione (GSH) activity [57,58,61,65]. These strains directly scavenge ROS, suppress lipid peroxidation, and improve oxidative stress parameters across multiple organ systems. Particularly, *Eubacterium rectale* synthesizes and secretes glutathione directly into the intestinal environment, providing immediate ROS scavenging capacity and limiting oxidative damage to both microbial communities and host tissues [141]. This represents a direct contribution of the microbiome toward enhancing the antioxidant capacity and maintaining redox homeostasis.

#### 5.1.4. Gut Microbiota-Redox-Immune Modulation

Next-generation probiotic candidates demonstrate sophisticated redox–immunomodulatory capabilities. *A. muciniphila*, often depleted in metabolic diseases, reduces systemic oxidative stress while suppressing pro-inflammatory cytokines, including TNF-α and IL-1β [68,70]. Similarly, *Parabacteroides distasonis* exerts multi-system protective effects, reducing oxidative stress and promoting anti-inflammatory immune responses. Even *P. copri*, despite its complex associations with autoimmune diseases, demonstrates neuroprotective effects through guanosine-mediated PI3K/Akt pathway activation, attenuating oxidative stress in post-traumatic brain injury models (Table 2) [142]. However, only a few small early-stage clinical studies have tested these strains in patients, and the findings remain preliminary. Larger, well-designed randomized controlled trials (RCTs) are required to validate these effects and determine their clinical significance.

#### 5.1.5. Considerations for Personalized Interventions 

The efficacy of probiotic interventions critically depends on baseline microbiota composition and individual host factors. Clinical studies reveal heterogeneous colonization patterns and transcriptional responses, with clear distinctions between responders and non-responders that can be predicted from baseline microbiome features [152]. When ecological niches are already occupied by resident strains, newly introduced probiotic bacteria colonize poorly and have limited therapeutic efficacy [153]. Furthermore, studies following antibiotic treatment demonstrate that empirical "one-size-fits-all" probiotic supplementation can actually delay both microbiome recovery and host transcriptome normalization [154]. These findings underscore the critical need for personalized probiotic selection based on individual microbiome profiling and functional capacity assessment. 

### 5.2. Microbiota-Mediated Biotransformation of Antioxidant Compounds

Many dietary antioxidants and phytochemicals require gut microbial biotransformation to exert their full redox-modulatory effects. Microbial deglycosylation, reduction, and ring fission can increase the bioavailability of these compounds and generate derivatives with stronger ROS-scavenging capacity, xanthine oxidase inhibition, or Nrf2–antioxidant response element (ARE) activation than the parent molecules [155,156,157,158]. Representative examples that meet these criteria are summarized in Supplementary Table S1 [159,160,161,162,163,164,165]. Consequently, inter-individual differences in microbiota composition and microbial enzymatic repertoires are likely to influence how antioxidant-based interventions effectively modulate the gut microbiota–redox–immune axis. At present, however, most of these data come from in vitro and animal models, and rigorously designed human trials are still needed before microbiota-dependent optimization of antioxidant therapies can be translated into clinical practice.

### 5.3. Baseline Microbiome-Informed Therapeutic Approaches

Recognition that baseline microbiome composition can substantially influence therapeutic responses has motivated the development of microbiota-stratified precision medicine strategies [166]. Rather than applying uniform interventions across populations, these approaches stratify individuals based on dominant microbial community patterns to optimize outcomes. However, most supporting evidence comes from relatively small, proof-of-concept studies.

**Figure 2:**
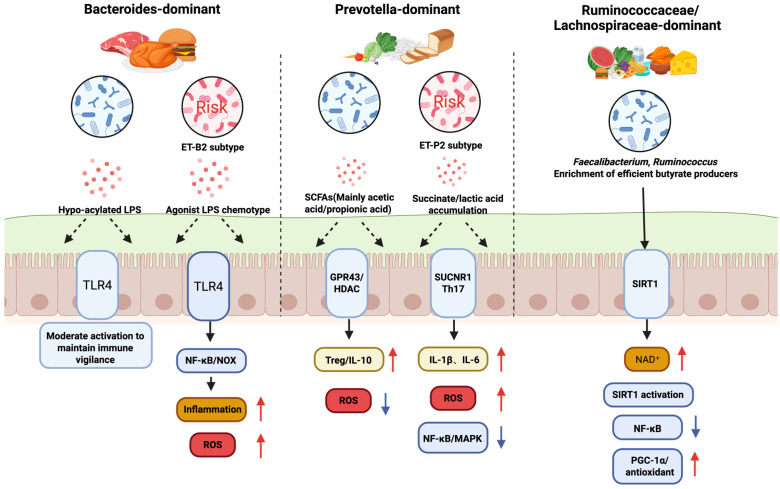
Microbial Community Configuration-Specific Regulation of Intestinal Redox and Immune Pathways. Red upward arrows denote increased levels, whereas blue downward arrows denote decreased levels.

Different microbial community configurations exhibit distinct metabolic capabilities that directly influence treatment responses (Figure 2). When provided with identical fiber substrates, *Prevotella*-enriched communities generate significantly higher total SCFA concentrations—particularly propionate—compared with *Bacteroides*-dominant communities [167]. These differences are associated with divergent therapeutic outcomes: high-fiber dietary interventions yield superior metabolic benefits in individuals with *Prevotella*-enriched configurations, whereas those with *Bacteroides*-dominant profiles may respond more favorably to strategies that selectively increase *Bifidobacterium* populations [168,169]. Such differential responses reflect the baseline metabolic capacities and inflammatory profiles associated with different community types. 

The *Prevotella*-to-*Bacteroides* (P/B) ratio has emerged as a quantitative metric for evaluating metabolic responses to dietary intervention. Research by Sandberg et al. demonstrated that while the glucose-lowering effects of barley dietary fiber were independent of baseline microbial composition, individuals with a high P/B ratio exhibited superior metabolic homeostasis, characterized by lower postprandial insulin levels, reduced systemic inflammation (IL-6), and diminished hunger sensations [170]. These findings suggest that baseline compositional metrics hold significant biomarker value for identifying underlying cardiometabolic risks and defining host inflammatory phenotypes, even when they do not fully predict interventional efficacy for specific substrates.

Microbiota-based stratification also influences clinical decision-making in inflammatory bowel disease (IBD) therapy. A prospective study by Canepeel et al. revealed that low-diversity *Bacteroides*-dominant configurations (Bact2 subtype)—characterized by reduced microbial load and strong association with inflammation—are prevalent among patients with active IBD, particularly those with ileal involvement [171]. The study demonstrated that baseline community composition significantly influences the efficacy of biologics, with patients harboring Bact2 configurations showing significantly higher response rates to first-line anti-tumor necrosis factor (anti-TNF) therapy compared to vedolizumab. Furthermore, anti-TNF treatment exhibited the capacity to remodel the gut microecology by increasing the abundance of butyrate-producing bacteria and restoring microbial load, thereby facilitating a transition from the dysbiotic Bact2 state toward a more balanced composition.

Community-based stratification has also shown promise in optimizing fecal microbiota transplantation (FMT). He et al. categorized FMT recipients as Enterobacteriaceae-dominant (RCPT/E) or *Bacteroides*-dominant (RCPT/B), revealing markedly different post-transplant engraftment patterns and clinical responses [172]. They further developed a colonization-to-recipient (C2R) index to quantify donor engraftment efficiency and an enterotype-based donor selection (EDS) model to improve donor–recipient matching. This represents a promising step beyond empirical FMT protocols toward rationalized donor selection. However, current microbiota-stratified FMT studies are limited to small cohorts and short follow-up, and their generalizability remains uncertain.

Importantly, microbial communities are better understood as compositional gradients rather than fixed categories, with most individuals occupying intermediate states. This implies that future microbiome-based interventions should consider continuous baseline characteristics—such as functional gene content, metabolic pathway enrichment, quantitative taxonomic ratios such as P/B ratio, or relative abundances of key functional guilds—rather than rely solely on discrete categorical labels. Compared with pharmacological therapies that directly target downstream inflammatory or metabolic pathways, microbiota-informed interventions remain largely conceptual and should currently be regarded as adjuncts rather than replacements for established treatments. Overall, microbiome- and redox-targeted strategies show encouraging preclinical and early clinical effects, but the translational gap to widespread clinical application remains substantial. Well-powered, rigorously controlled randomized trials with standardized redox and clinical endpoints will be essential before these approaches can be incorporated into routine clinical practice.

This figure illustrates how different gut microbial community configurations, defined by their dominant bacterial species and influenced by diet, distinctly regulate intestinal health, particularly in relation to oxidative stress and immune responses. The *Bacteroides*-dominant community, often associated with a high-protein diet, can produce hypo-acylated LPS or agonist LPS. These activate the TLR4 pathway, leading to moderate immune activation and maintaining immune vigilance, but can also contribute to inflammation and increased ROS. The *Prevotella*-dominant community, linked to a high-carbohydrate diet, produces large amounts of SCFAs and succinate/lactic acid. While SCFAs activate GPR43 to promote anti-inflammatory responses (Treg/IL-10), succinate can stimulate the SUCNR1 pathway, leading to increased ROS and inflammation (IL-1β, IL-6, NF-κB/MAPK). Lastly, the *Ruminococcus*/*Lachnospira*-enriched community is characterized by efficient butyrate producers like *Faecalibacterium* and *Ruminococcus*. Butyrate provides energy for the host and activates the SIRT1 pathway via NAD+, which downregulates inflammatory responses (NF-κB) and upregulates antioxidant pathways (PGC-1α), promoting a healthy, low-stress state, free fatty acid receptor 2/3; SUCNR1, succinate receptor 1; Th17, T helper 17 cells; MAPK, mitogen-activated protein kinase; PGC-1α, peroxisome proliferator-activated receptor-γ coactivator-1α; NAD^+^, nicotinamide adenine dinucleotide, oxidized form; IL-1β, interleukin-1 beta, NF-κB, Nuclear Factor kappa-light-chain-enhancer of activated B cell, MAPK, mitogen-activated protein kinase.

## 6. Challenges and Prospects for Personalized Microbiome Therapy

The major limitations of the current evidence and future directions were summarized, explicitly distinguishing well-supported findings from more speculative, hypothesis-generating aspects of the proposed microbiota–redox axis framework. The implementation of personalized microbiome therapeutics faces significant challenges due to substantial inter-individual variability in treatment responses. This variability reflects the complex interplay of host genetics, demographics such as age and sex, environmental exposures including diet, medications, and lifestyles, and baseline microbiome composition, all of which collectively shape intervention outcomes. Addressing and understanding these sources of heterogeneity represent critical frontiers for advancing precision microbiome medicine.

### 6.1. Sources of Therapeutic Heterogeneity and Predictive Biomarkers

Baseline microbiome features, including α-diversity, core taxonomic composition, and functional metabolic potential, serve as key predictors of treatment responses, enabling identification of likely responders versus non-responders [173]. Clinical studies demonstrate clear mechanistic differences between these groups. Following *Lacticaseibacillus casei* (*L. casei*) Zhang administration, responders exhibit increased beneficial taxa such as *Roseburia* species and downregulated LPS biosynthesis pathways, resulting in reduced systemic oxidative stress markers. Non-responders lack these beneficial shifts, likely due to colonization resistance from established microbial communities [174].

FMT is characterized by similar heterogeneity in response across therapeutic applications. In a randomized, double-blind, placebo-controlled trial evaluating FMT for Parkinson’s disease, a distinct subset of patients showed marked clinical improvements, including decreased Unified Parkinson’s Disease Rating Scale (UPDRS) scores and rapid relief of motor and non-motor symptoms such as constipation [175]. However, other participants demonstrated minimal changes, creating substantial outcome variability that inflated group means but obscured individual-level therapeutic potential.

The mechanistic basis for heterogeneity in FMT response likely involves recipient intestinal environments that vary in their permissiveness to donor microbiome engraftment and capacity for functional “redox rebalancing” [176]. Responders possess gut ecological conditions conducive to the establishment and metabolic integration of donor strains. Non-responders exhibit colonization resistance or fail to achieve the functional microbiome shifts necessary for therapeutic benefit. This understanding emphasizes the critical importance of pre-treatment assessment of recipient "plasticity" and development of function-matched donor selection protocols. 

### 6.2. Community-Based Stratification Approaches

**Figure 3:**
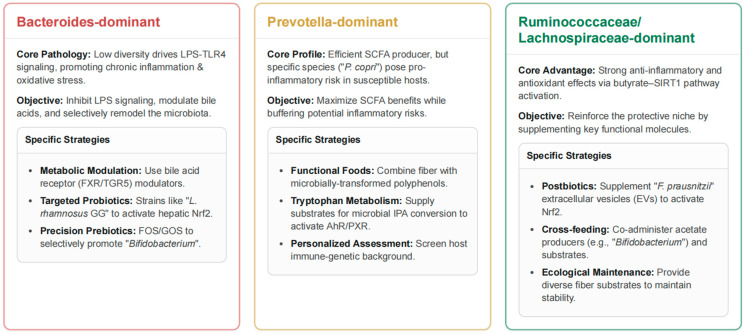
Community Configuration-Targeted Strategies for Microbiome Intervention.

Microbial community stratification offers a practical, albeit simplified, method for categorizing individuals based on community-level functional tendencies rather than focusing solely on individual bacterial taxa [34]. Among commonly discussed community configurations, *Prevotella*-enriched and *Bacteroides*-dominant communities display distinct metabolic, redox-regulatory, and inflammatory profiles, although they exist along a continuous compositional spectrum rather than as strictly discrete states [177]. *Prevotella*-enriched communities are associated with fiber-rich, plant-based dietary patterns and demonstrate efficient fermentation of complex carbohydrates, producing SCFAs with particular emphasis on propionate [169]. Butyrate, a key SCFA, functions as a histone deacetylase inhibitor, activating Nrf2-mediated antioxidant pathways while suppressing NF-κB–driven inflammatory signaling [178]. Figure 3 summarizes principles for community-specific interventions. 

Despite these benefits, *Prevotella*-enriched communities also exhibit potential pro-inflammatory characteristics. Predominant *Prevotella* species, especially *P. copri*, can trigger inflammatory responses and have been linked to increased incidence of rheumatoid arthritis in genetically susceptible individuals [51,52]. The inflammatory potential of these communities is context-dependent, shaped by species composition, host immune genetics, and concurrent dietary factors. This duality highlights the complex role of *Prevotella* species, which can act as both beneficial fiber-fermenters producing anti-inflammatory SCFAs and as potential inflammatory triggers. Mechanisms underlying the pro-inflammatory aspects may include enhanced antigen presentation, increased production of inflammatory metabolites, or promotion of Th17-skewed immune responses, contributing to autoimmune pathology.

*Bacteroides*-dominant communities are typically associated with high-protein, high-fat Western dietary patterns and display distinct metabolic specializations, including bile acid transformations (deoxycholic acid and lithocholic acid production) and protein/fat metabolism pathways that generate branched-chain amino acids [179,180,181]. Although *Bacteroides*-enriched configurations are traditionally considered pro-inflammatory due to LPS production, some *Bacteroides* species synthesize hypo-acylated LPS with reduced TLR4 activation, resulting in a more moderate inflammatory profile than previously recognized [9]. Pathological *Bacteroides*-dominant configurations (Bact2 subtype) are strongly linked to obesity and systemic inflammation, characterized by low microbial diversity and depletion of protective butyrate-producing species such as *F. prausnitzii* [134]. These observations underscore the importance of species-level resolution within community types and the consideration of host-specific factors in designing therapeutic strategies. For individuals with *Prevotella*-enriched communities, interventions should leverage the SCFA-producing capacity while mitigating pro-inflammatory risks, particularly in those with autoimmune predispositions. For those with *Bacteroides*-dominant configurations, particularly the Bact2 subtype, strategies should aim to promote beneficial microbial species while preserving the protective features of specific *Bacteroides* strains [182]. 

This figure outlines specific, community-driven therapeutic strategies for managing the gut microbiome to improve host health. For *Bacteroides*-dominant communities, the core issue is low microbial diversity driving chronic inflammation and oxidative stress via LPS-TLR4 signaling. The objective of the intervention is to inhibit LPS, modulate bile acids, and remodel the microbiota using strategies like metabolic modulators such as FXR/TGR5 agonists and targeted probiotics to promote *Bifidobacterium* and other beneficial strains. *Prevotella*-dominant communities are efficient SCFA producers, but certain species like *Prevotella (P.) copri* can pose an inflammation risk. The strategy here is to maximize the benefits of SCFAs while buffering these risks. This is achieved through functional foods combining fiber with microbially transformed polyphenols and supplying substrates for tryptophan metabolism. Finally, the *Ruminococcus/Lachnospira*-enriched community configuration is advantageous due to its strong anti-inflammatory and antioxidant effects via the butyrate-SIRT1 pathway. The objective is to reinforce this protective niche using strategies like postbiotics, such as supplementing *Faecalibacterium* (*F.*) *prausnitzii* extracellular vesicles, cross-feeding (co-administering acetate producers with substrates), and providing diverse fiber sources to maintain ecological stability. LPS, lipopolysaccharide; TLR4, Toll-like receptor 4; FXR/TGR5, farnesoid X receptor/Takeda G-protein–coupled bile-acid receptor 5; *L. rhamnosus* GG, *Lacticaseibacillus rhamnosus* strain GG; Nrf2, nuclear factor erythroid 2–related factor 2; FOS/GOS, fructo-/galacto-oligosaccharides; SCFAs, short-chain fatty acids; IPA, indole-3-propionic acid; AhR/PXR, aryl-hydrocarbon receptor/pregnane X receptor; SIRT1, sirtuin-1; EVs, extracellular vesicles.

### 6.3. Advanced Methodological Approaches for Establishing Causality

#### 6.3.1. Multi-Omics Integration and Elucidation of Mechanistic Pathways

Observational microbiome studies face substantial confounding from dietary patterns, pharmaceutical exposures, host genetics, and reverse causation, limiting causal inference capabilities [183,184,185]. A systematic approach to address these limitations involves translating population-level associations into testable molecular mechanisms through comprehensive multi-omics integration encompassing metagenomics, metatranscriptomics, and metabolomics [186]. This integrated approach provides complementary insights: metagenomics delineates taxonomic composition and functional genetic potential, metatranscriptomics captures active microbial and host gene expression patterns, and metabolomics resolves the actual bioactive compounds and metabolic pathways operative *in vivo*. Systematic integration across these molecular layers generates mechanistically grounded hypotheses that connect specific microbial taxa through key functional genes to effector metabolites and ultimately to pathophysiological outcomes in the host [186]. 

#### 6.3.2. Mendelian Randomization for Causal Inference

These mechanistic hypotheses can be rigorously tested for causal directionality using Mendelian randomization (MR) approaches, which leverage randomly assorted genetic variants as instrumental variables to infer causal relationships between exposures (specific microbes or metabolites) and health outcomes [183,187]. MR can systematically evaluate whether the abundance of particular bacterial taxa causally influences specific metabolite concentrations, supporting complete causal chains of the form "specific microbe → metabolite → disease outcome via known adaptive or maladaptive metabolite pathway." Current MR applications in microbiome research face methodological challenges, including weak instrumental variables and limited taxonomic resolution [188]. However, advancing technologies, including whole-genome shotgun metagenomics, larger population cohorts, and sophisticated analytical methods, are rapidly improving the feasibility and statistical power of causal inference approaches [189].

### 6.4. Future Directions: Toward Continuous Microbiome Metrics and Predictive Modeling

#### 6.4.1. Evolution Beyond Discrete Community Classifications

Accumulating evidence indicates that microbial community configurations represent points along a continuous spectrum rather than discrete, sharply partitioned categories, with Ruminococcaceae-enriched patterns often appearing as transitional states between *Bacteroides*- and *Prevotella*-dominant communities [34]. This recognition has motivated the development of continuous metrics such as *Bacteroides/Prevotella* ratios, which often outperform traditional three-class categorizations in predicting therapeutic outcomes. Clinical validation of this approach comes from a 26-week dietary intervention study, demonstrating that higher *Prevotella/Bacteroides* ratios predicted greater fat loss responses to high-fiber New Nordic Diet interventions [177]. Extending continuous taxonomic ratio approaches to predict responses across diverse therapeutic modalities, including specific diets, prebiotic supplements, probiotic strains, and FMT protocols, represents a logical and promising next step for precision microbiome medicine.

#### 6.4.2. Expanding Beyond Traditional Community Paradigms

Conventional community stratification frameworks have primarily focused on *Bacteroides*- and *Prevotella*-dominant distinctions, with less emphasis on Ruminococcaceae- or Lachnospiraceae-enriched community states. Emerging evidence indicates that Ruminococcaceae/Lachnospiraceae-enriched communities may be associated with more favorable microbial and host profiles across multiple disease contexts, although the current data are limited and largely observational.

In ulcerative colitis, Lachnospiraceae-enriched patterns are linked to reduced abundance of the pathogenic *Ruminococcus gnavus* and stronger associations with beneficial taxa such as *Odoribacter splanchnicus* and *Bacteroides uniformis*, suggesting intrinsic anti-inflammatory properties [44]. Similarly, in studies of depression, Lachnospiraceae-enriched states differentiate patients from healthy controls at the β-diversity level and correlate with favorable metabolic, neurotransmitter, and immune pathway profiles, highlighting a potential modulatory role in mental health. These findings point to the potential utility of family-level taxonomic ratios, encompassing Bacteroidaceae, Lachnospiraceae, and Prevotellaceae, as candidate predictive biomarkers for disease susceptibility and therapeutic optimization. However, their predictive accuracy, temporal stability, and generalizability across diverse populations and sequencing platforms require rigorous validation before clinical application.

#### 6.4.3. Advanced Study Designs for Development of Personalized Interventions

N-of-1 experimental designs, where individual participants cycle through multiple randomized intervention periods with appropriate washout phases, offer particular advantages for personalized nutrition and microbiome studies [190]. These designs enable within-person mapping from continuous microbiome metrics (such as *Prevotella*/*Bacteroides* ratios, *Bacteroides*/*Lactobacillus* ratios, and family-level taxonomic proportions) to functional and metabolic endpoints, including SCFA profiles, bile acid compositions, TMAO levels, indole-3-propionate concentrations, and composite redox/antioxidant indices. Aggregating data from multiple N-of-1 trials provides high-quality longitudinal training datasets for machine learning models that can leverage microbiome time series data to identify features predictive of changes in host physiological state, ultimately enabling the development of generalizable personalized intervention algorithms.

#### 6.4.4. Machine Learning Applications and Advances in Predictive Modeling 

Recent studies highlight the increasing sophistication and translational potential of microbiome-based predictive modeling. For example, a random forest classifier trained on quantitative microbiome features from 21,561 individuals accurately distinguished vegan, vegetarian, and omnivore dietary patterns across multiple independent datasets, demonstrating that diet–microbiome relationships can be effectively captured and generalized through machine learning approaches [191,192]. In controlled intervention studies, Bioorthogonal Non-Canonical Amino Acid Tagging–Fluorescence-Activated Cell Sorting (BONCAT-FACS)-informed random forest models successfully predicted individual postprandial satiety responses to supplementation with high-dose corn-bran arabinoxylan versus microcrystalline cellulose. Interestingly, while microbial taxonomic composition alone offered limited explanatory power for improvements in insulin resistance and inflammatory markers, changes in bile acid metabolites provided superior predictive capability [193]. These findings emphasize the importance of substrate-specific metabolite profiling and functional guild analysis in microbiome-based predictions [194].

**Figure 4:**
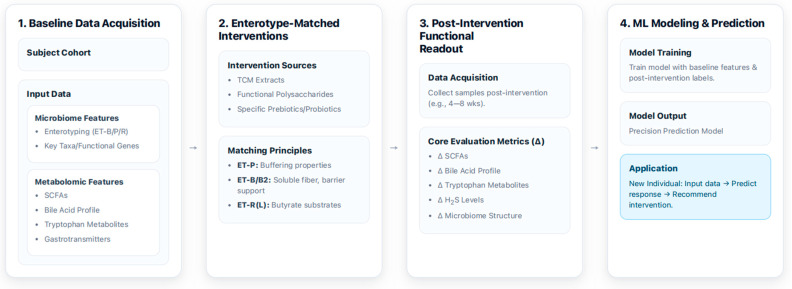
A Machine Learning Framework for Precision Microbiome Intervention.

Functional genomic approaches further enhance predictive accuracy. By combining ex vivo stool enrichment with metagenomic analysis, researchers identified substrate-specific carbohydrate gene clusters containing carbohydrate-active enzymes (CAZymes), transporters, and regulatory elements for processing xylo-oligosaccharides (XOS), fructo-oligosaccharides (FOS), and inulin (Figure 4). Training classification algorithms using SCFA profiles as response phenotypes accurately identified human responders to specific prebiotic substrates in independent validation cohorts, illustrating that functional genetic content can provide more precise guidance for personalized polysaccharide interventions than taxonomic composition alone [195]. The Metabolite Response Predictor using Coupled Multilayer Perceptrons (McMLP) framework represents a notable methodological advance, leveraging baseline microbiome profiles to predict metabolomic changes following dietary interventions through multi-task deep learning. This framework demonstrates promising cross-study and cross-food response prediction capabilities, moving toward truly personalized nutrition recommendations [196]. 

Despite these advances, several challenges remain in microbiome-based machine learning. Large datasets often introduce batch effects, inconsistencies in sample collection and data entry, and variability in preprocessing pipelines, all of which can embed systemic noise before model training. Conversely, small datasets increase the risk of overfitting and reduce generalizability to independent populations. Additionally, many algorithms still operate as “black boxes,” limiting mechanistic interpretability. For clinical translation, models must undergo rigorous validation across independent cohorts and real-world conditions to ensure reliable predictive performance.

It illustrates a machine learning-based framework for developing precision interventions targeting the gut microbiome. The process begins with baseline data acquisition, where a subject cohort is analyzed to collect detailed information on their microbiome, such as community configuration, key taxa, and metabolome, such as SCFAs and bile acids. Next, community-matched interventions are applied, utilizing specific prebiotics/probiotics or TCM extracts based on the individual’s microbial community configuration to achieve targeted effects. *Prevotella*-dominant communities (ET-P) are matched with buffering properties, *Bacteroides*-dominant communities with barrier support, and *Ruminococcus*/*Lachnospira*-enriched communities (ET-R/L) with butyrate substrates. Following the intervention, a post-intervention functional readout collects new data and evaluates core metrics like changes in SCFA levels, bile acid profiles, and microbiome structure. Finally, this comprehensive dataset is used for ML modeling and prediction. The model is trained to learn from the baseline and post-intervention data, creating a precision prediction model that can then be applied to new individuals. By inputting a new subject’s baseline data, the model can predict their response to a specific intervention and recommend a personalized strategy. ET-B2, low-diversity *Bacteroides* subtype; SCFAs, short-chain fatty acids; TCM, traditional Chinese medicine; H_2_S, hydrogen sulfide; ML, machine learning; Δ (delta), change from baseline.

#### 6.4.5. Integration Toward Implementation of Precision Medicine

Future advances in microbiome-based precision medicine will require systematic integration of continuous microbiome metrics with N-of-1 experimental designs to monitor temporal changes in microbial community composition and metabolic outputs following diverse interventions, including dietary modifications, probiotic supplementation, and prebiotic administration [190]. Longitudinal studies incorporating comprehensive multi-omics endpoints—such as SCFA profiles, bile acid spectra, tryptophan metabolites, and gaseous signaling molecules—will be essential for establishing dynamic relationships between community composition and host metabolic health outcomes. Such data can provide more robust training and validation datasets for advanced machine learning models.

In principle, long-term, multi-timepoint predictive frameworks that integrate microbial composition, metabolomics, and functional readouts could support the development of generalizable, individualized nutrition and microbiome intervention strategies. However, several challenges remain: controlling batch effects, minimizing overfitting, validating models in independent cohorts, and improving the interpretability of algorithmic predictions are all critical to translating these approaches into clinically actionable tools.

### 6.5. Current Challenges and Future Directions

Despite rapid progress, the evidence linking the gut microbiota, oxidative stress, and disease remains fragmentary and largely associative. Most data derive from cross-sectional cohorts, short-term trials, or preclinical models, and associations between specific taxa or metabolites and clinical outcomes often weaken after adjustment for diet, medications, and other confounders. Conceptually, microbial community stratification provides a practical framework to describe recurrent community configurations, but these exist along a compositional continuum and exhibit strong context dependence. Therefore, current microbiota–redox models should be viewed as hypothesis-generating rather than definitive.

Methodological limitations—including heterogeneity in sequencing and analytical pipelines, overfitting, and limited external validation of multi-omics machine learning models—further constrain causal inference. Moving forward, priorities include: (i) conducting larger longitudinal and interventional studies with standardized redox and immune endpoints; (ii) deepening mechanistic investigations using gnotobiotic and ex vivo systems; and (iii) developing more interpretable and externally validated multi-omics frameworks. These efforts will be essential to transform the proposed microbiota–redox axis into a robust, evidence-based foundation for precision nutrition and microbiome-targeted therapeutic strategies.

## 7. Conclusions

Oxidative stress is a key pathway through which environmental exposures, diet, and the gut microbiota jointly influence metabolic, cardiovascular, and neurodegenerative diseases. Microbiota-derived metabolites—including SCFAs, secondary bile acids, tryptophan derivatives, and gasotransmitters—modulate a gut redox–immune axis that shapes barrier integrity and immune responses in metabolic organs. Microbial community stratification provides a useful but still largely conceptual framework for organizing these interactions, as direct interventional or longitudinal evidence linking *Bacteroides-*, *Prevotella*-, *Bifidobacterium-*, *Proteobacteria-*, *Ruminococcus-* and *Lachnospira*-dominant profiles to oxidative stress markers, clinical outcomes, or therapeutic responses remains scarce. Accordingly, microbiota–redox relationships should at present be regarded as hypothesis-generating, and future work should prioritize: (1) standardized and reproducible community profiling; (2) longitudinal tracking of community composition and redox dynamics; and (3) multi-omics integration to define causal pathways that can be tested in targeted intervention trials.

## Figures and Tables

**Table 1:** Probiotic Bacteria and Their Redox-Modulatory Mechanisms in Therapeutic Applications.

Probiotic Bacteria Genus	Author (et al.)	Target Microbe	Mechanism	Study Model
*Lactobacillus (L.)*	Ahire et al. [56]	*L. helveticus CD6*	Produces antioxidant folate; Metal ion chelation	Cell
	Wang et al. [57]	*L. plantarum (CCFM1149/10)*	↑Host antioxidant enzymes (CAT, SOD)	Cell
	Noureen et al. [58]	*L. brevis MG000874*	Direct ROS scavenging; ↑Host antioxidant enzymes	Animal
	Marsova et al. [59]	*L. fermentum U-21*	Metal chelation; ↑GSH and GPx levels	Animal
	Kamaladevi et al. [60]	*L. casei*	↑DAF-16/FOXO pathway; ↑SOD, GSH	Animal
	Miraghajani et al. [61]	*L. plantarum A7*	↑Intracellular glutathione (GSH) pathway	Clinical
	Saeedi et al. [62]	*L. rhamnosus GG*	↑Hepatic Nrf2 signaling pathway	Cell, Animal
	Liu et al. [63]	*L. johnsonii JJB3*	↑BNIP3L-mediated mitophagy	Cell, Animal
	Bell et al. [64]	*L. reuteri*	Alters redox balance; ↓Protein translation	Cell, Animal, Human
*Bifidobacterium (B.)*	Wang et al. [57]	*B. longum CCFM752*	↑Host antioxidant enzymes (CAT, SOD)	Cell
	Bernier et al. [65]	*B. breve MCC1274*	Produces niacin; ↓Lipid droplet-related gene (PLIN4)	Cell
	Shen et al. [66]	*B. animalis 01*	Direct free radical scavenging; ↑Host antioxidant enzymes	Animal
	Soleimani et al. [67]	*B. bifidum*	↑KEAP1-Nrf2 pathway; ↑Total antioxidant capacity	Animal + Human
Other Key Species	Zhang et al. [68]	*Akkermansia muciniphila*	↓Oxidative stress and inflammation; Modulates gut microbiota	Animal
	Desaka et al. [69]	*Streptococcus thermophilus*	↑DAF-16/FOXO pathway	Animal
	Wei et al. [70]	*Parabacteroides distasonis*	↑SOD2; ↓NF-κB signaling pathway	Animal
	Cao et al. [71]	*Enterococcus faecium*	Intrinsic antioxidant activity; ↑Host serum SOD	Animal
	Ye et al. [72]	*Faecalibacterium prausnitzii*	↑Nrf2/HO-1 pathway; ↓NF-κB	Cell + Animal
	Li et al. [73]	*Clostridium butyricum*	↑Nrf2 signaling pathway	Cell + Animal

Upward arrows denote increased levels, whereas downward arrows denote decreased levels. BNIP3L, BCL2/adenovirus E1B 19 kDa protein-interacting protein 3-like; CAT, catalase; SOD, superoxide dismutase; Nrf2, nuclear factor erythroid 2-related factor 2; KEAP1, Kelch-like ECH-associated protein 1; PLIN4, perilipin 4; NF-κB, nuclear factor kappa-light-chain-enhancer of activated B cells; HO-1, heme oxygenase-1; FOXO, forkhead box transcription factors class O; SOD2, superoxide dismutase-2.

**Table 2:** Key Molecular Mediators of the Microbiota–Redox–Immune Axis in metabolic diseases.

Molecule	Source	Primary Function/Effect	Specific Mechanism	Pathophysiological Role
LPS	Gut Microbiota (*Bacteroides*, *Prevotella*-2)	Pro-inflammatory	Translocates through leaky gut, activates inflammatory pathways [143]	Induces metabolic endotoxemia and systemic inflammation, leading to insulin resistance. Contributes to MASLD by triggering liver inflammation [144,145].
SCFAs	Gut Microbiota (Commensals)	Anti-inflammatory, Metabolic	Inhibit HDACs, activate GPR109a, promote Treg cell development, and provide energy for colonocytes [146]	Improve insulin sensitivity, reduce inflammation. Strengthen gut barrier. Increase energy harvest, contributing to obesity [139,147].
BCAAs	Diet, Gut Microbiota (*Bacteroides*)	Pro-insulin resistance	Activate mTORC1/S6K1, which phosphorylates and inactivates IRS-1 [135,136]	High plasma levels are strongly associated with obesity, insulin resistance, and T2DM [136].
TMAO	Gut Microbiota (*Bacteroides*)	Pro-atherosclerotic	May alter cholesterol metabolism, inflammation, and endothelial function [148]	Correlated with an increased risk of atherosclerotic CVD [148].
TNF-α	Host (Adipose tissue, macrophages)	Pro-inflammatory	Activates JNK and IKKβ/NF-κB pathways, which phosphorylate IRS-1 [149]	Impairs insulin signaling and glucose uptake, a central mediator of metaflammation-induced insulin resistance [150].
ROS/RNS	Host (NOX enzymes), Gut Microbiota	Dual role: Signaling and Damage	Oxidize redox-sensitive proteins, affecting pathways like MAPK and NF-κB [25]	High levels exacerbate oxidative stress and cellular damage; controlled levels are crucial for immune activation and signaling. A vicious cycle with dysbiosis exacerbates liver damage in MASLD [151].

LPS, lipopolysaccharide; SCFAs, short-chain fatty acids; BCAAs, branched-chain amino acids; TMAO, trimethylamine N-oxide; TNF-α, tumor necrosis factor-alpha; ROS, reactive oxygen species; RNS, reactive nitrogen species; HDACs, histone deacetylases; GPR109a, G-protein coupled receptor 109A; mTORC1, mechanistic target of rapamycin complex 1; S6K1, ribosomal protein S6 kinase beta-1; IRS-1, insulin receptor substrate-1; JNK, c-Jun N-terminal kinase; IKKβ, IκB kinase beta; NF-κB, nuclear factor kappa-light-chain-enhancer of activated B cells; MAPK, mitogen-activated protein kinase; T2DM, type 2 diabetes; CVD, cardiovascular disease; MASLD, metabolic dysfunction-associated steatotic liver disease.

## Data Availability

No new data were created or analyzed in this study. Data sharing is not applicable to this article.

## Supplementary Materials

The following supporting information can be downloaded at https://www.mdpi.com/article/10.3390/antiox15020175/s1: Table S1. Gut Microbiota-Mediated Biotransformation of Antioxidant Compounds and Enhanced Bioactivity.
