# Supplementary material for: Gut Microbial Composition, Oxidative Stress, and Immunity in Metabolic Disease: Toward Personalized Interventions
Source: Antioxidants (Basel). 2026 Jan 29;15(2):175. doi: 10.3390/antiox15020175 (PMC12938183; doi:10.3390/antiox15020175)
Supplement: Supplementary file 1 [file antioxidants-15-00175-s001.zip › antioxidants-4098146-supplementary.pdf]

Table S1. Gut Microbiota-Mediated Biotransformation of Antioxidant Compounds and Enhanced Bioactivity

| Authors | Parent Compound | Drug Type | Gut Microbial Metabolite | Metabolic Reaction Type | Evidence of Enhanced Antioxidant Activity | Mechanism of Action |
|---|---|---|---|---|---|---|
| Wang et al.[156] | Cyanidin-3-O-β-glucoside | Polyphenol/ Anthocyanin | PCA | Deglycosylation, C-ring fission | At physiological concentrations, PCA shows significant anti-atherosclerotic activity; the parent compound is ineffective | Inhibits NF-κB pathway, reduces expression of adhesion molecules (VCAM-1, ICAM-1); promotes cholesterol efflux. |
| Dong et al. [159] | Baicalin | TCM/Flavone | Baicalein | Deglycosylation, deglucuronidation | Baicalein has stronger xanthine oxidase inhibitor ($IC_{50}$ nearly 70-fold lower) than baicalin; activates the Nrf2 pathway. | Inhibits superoxide anion production; activates endogenous antioxidant defense system. |
| Di Meo et al. [160] | Curcumin | Polyphenol/ Curcuminoid | THC, OHC | Reduction | THC and OHC exhibit superior anti-inflammatory activity compared to curcumin, more effectively inhibiting COX-2 and NF-κB pathways. | Stronger anti-inflammatory effects, indirectly reducing inflammation-associated oxidative stress. |
| Zheng et al. [161] | Ferulic acid (FA) | Polyphenol/ Phenolic acid | 3,4diOHPPA | Conversion | 3,4diOHPPA has stronger antioxidant and antiproliferative effects than FA; induces apoptosis in cancer cells nearly 5 times more effectively than FA. | Stronger direct antioxidant, antiproliferative, and pro-apoptotic activity. |
| Sharma and Lee [162] | Ginsenoside Rb1 | TCM/Saponin | Ginsenoside, CK | Deglycosylation | CK shows higher bioactivity and bioavailability than the parent ginsenoside, with stronger anti-inflammatory effects (inhibition of NO and pro-inflammatory cytokines). | Stronger anti-inflammatory activity and immune regulation. |

| | | | | | | |
|---|---|---|---|---|---|---|
| Wang et al. [163] | Icariin | TCM/Flavonoid glycoside | Icaritin | Deglycosylation | Icaritin possess superior pharmacological activity compared to icariin, with stronger neuroprotective and antioxidative effects. | Higher bioavailability and stronger direct cytoprotective effects. |
| Nascimento et al. [164] | Glycyrrhizin | TCM/Triterpenoid saponin | GA | Deglycosylation; deglucuronidation | In DPPH radical scavenging assay, GA showed significantly stronger antioxidant activity than glycyrrhizin (at 1000 µg/100 µL, scavenging rate 76.85% vs 41.41%). | Stronger direct radical scavenging ability. |
| Desreumauxet al. [165] | Sulfasalazine | Synthetic Drug | 5-ASA | Azo-bond reduction | Sulfasalazine is an inactive prodrug; all therapeutic effects come from colonic release of 5-ASA. | Acts as a potent free radical scavenger, directly neutralizing multiple ROS and RNS; provides local anti-inflammatory and antioxidant effects. |

PCA, protocatechuic acid; THC, tetrahydrocurcumin; OHC, Octahydrocurcumin; 3,4diOHPPA, 3-(3,4-dihydroxyphenyl)propionic acid; GA, glycyrrhetinic acid; 5-ASA, 5-aminosalicylic acid; CK, Compound K; NRF2, nuclear factor erythroid 2-related factor 2; NF-κB, nuclear factor kappa-light-chain-enhancer of activated B cells; NO, nitric oxide; COX-2, cyclooxygenase-2; VCAM-1, vascular cell adhesion molecule-1; ICAM-1, intercellular adhesion molecule 1; ROS, reactive oxygen species; RNS, reactive nitrogen species; TCM, traditional Chinese Medicine.
